# Initial validation of a simulation model for estimating the impact of serogroup A *Neisseria meningitidis* vaccination in the African meningitis belt

**DOI:** 10.1371/journal.pone.0206117

**Published:** 2018-10-25

**Authors:** Michael L. Jackson, Alpha Oumar Diallo, Isaie Médah, Brice Wilfried Bicaba, Issaka Yaméogo, Daouda Koussoubé, Rasmata Ouédraogo, Lassané Sangaré, Sarah A. Mbaeyi

**Affiliations:** 1 Kaiser Permanente Washington Health Research Institute, Kaiser Permanente Washington, Seattle, Washington, United States of America; 2 National Center for Immunization and Respiratory Diseases, Centers for Disease Control and Prevention, Atlanta, Georgia, United States of America; 3 Direction de la Lutte Contre la Maladie, Ministère de la Santé, Ouagadougou, Burkina Faso; Public Health England, UNITED KINGDOM

## Abstract

We previously developed a mathematical simulation of serogroup A *Neisseria meningitidis* (NmA) transmission in Burkina Faso, with the goal of forecasting the relative benefit of different vaccination programs. Here, we revisit key structural assumptions of the model by comparing how accurately the different assumptions reproduce observed NmA trends following vaccine introduction. *A priori*, we updated several of the model’s parameters based on recently published studies. We simulated NmA disease under different assumptions about duration of vaccine-induced protection (including the possibility that vaccine-induced protection may last longer than natural immunity). We compared simulated and observed case counts from 2011–2017. We then used the best-fit model to forecast the impact of different vaccination strategies. Our updated model, with the assumption that vaccine-induced immunity lasts longer than immunity following NmA colonization, was able to reproduce observed trends in NmA disease. The updated model predicts that, following a mass campaign among persons 1–29 years of age, either routine immunization of 9 month-old children or periodic mini-campaigns among children 1–4 years of age will lead to sustained control of epidemic NmA in Burkina Faso. This validated model can help public health officials set policies for meningococcal vaccination in Africa.

## Introduction

Countries in the “meningitis belt” in sub-Saharan Africa experience the highest known incidence of meningococcal meningitis in the world. Countries in the meningitis belt experience periodic epidemics of *Neisseria meningitidis* with annual incidence rates exceeding 250 cases per 100,000 persons.[1, 2] Historically, major epidemics occurred every seven to ten years, and approximately 90% of cases during epidemics were caused by serogroup A *N*. *meningitidis* (NmA).[3–5] In response to this burden of disease, a novel serogroup A polysaccharide-tetanus toxoid conjugate vaccine (PsA-TT, MenAfriVac) was developed for use in the meningitis belt.[6, 7] To date, PsA-TT vaccination campaigns targeting persons 1–29 years of age have been conducted in 21 countries.These campaigns have been followed by 90% or greater reductions in NmA incidence, leading to the hope that PsA-TT vaccination programs can change the epidemiology of meningococcal disease in Africa.[8–10]

Long-term vaccination strategies are now needed to maintain the successful reductions in disease incidence achieved by the campaigns. Many potential long-term vaccination strategies exist, including periodic catch-up campaigns among selected age groups or addition of PsA-TT to the Expanded Programme on Immunization (EPI) schedule. Mathematical models can help policy makers identify the most effective vaccination strategies by forecasting the expected impact of different vaccination strategies under various assumptions. Towards this end, we previously developed a mathematical model of NmA in the meningitis belt and used it to forecast the impact of PsA-TT vaccination strategies in Africa.[11] Our model was developed based on data available prior to the first introduction of PsA-TT in Burkina Faso in 2010. At that time, limited data were available about the expected duration of vaccine-induced protection against NmA colonization and disease. Due to the limited data, we used two different assumptions about the duration of protection following PsA-TT. Our “Base” model assumed that PsA-TT protection was equivalent to natural immunity following colonization or disease, while our “Vaccination-Plus” model assumed that immunity following PsA-TT lasted longer than natural immunity.

Now that several years have elapsed following introduction of PsA-TT in Burkina Faso, we revisit our model to see whether the Base or Vaccination-Plus assumption is more consistent with observed NmA incidence. We then compare expected impact of different vaccination strategies over a 50-year time horizon.

## Methods

### Model structure, parameters, and initial conditions

**Fig 1:**
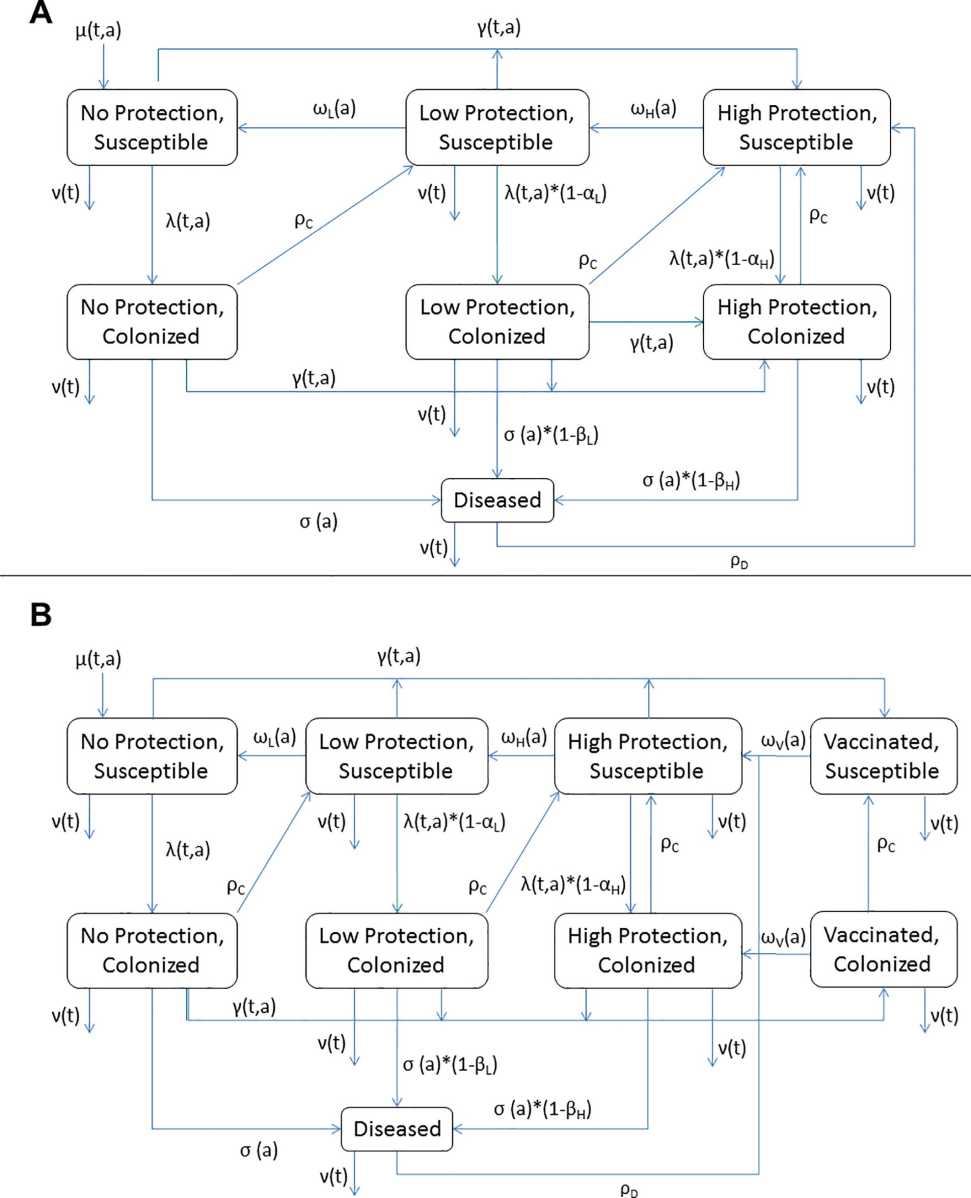
**Model structure for Base (A) and Vaccination-Plus (B) models**. Each figure denotes movement within a given age stratum (aging process not shown). μ, birth rate; ν, death rate; γ, vaccination rate; other symbols as in Table 1. Subscripts t and a indicate parameters that vary with calendar time or age, respectively.

Details of the model and population have been previously published.[11] In brief, we developed an age-structured compartmental model that partitions the population into mutually exclusive states based on age, infection status (susceptible, colonized, or diseased), and level of protection against NmA colonization and disease (High, Low, or None) (Fig 1A). The use of High and Low protection states allows immunity against invasive disease to persist longer than immunity against asymptomatic colonization with NmA in the model, consistent with observations about the duration of immunity based on serum bactericidal antibodies (SBA) concentrations.[12]

**Table 1:** Model parameters. Symbols correspond to symbols used in Fig 1. All units except percents are years^-1^. Original values were used for the Original Base and Original Vaccination-Plus models. Prior values indicate the range used in updating the model through approximate Bayesian computing (ABC). Posterior means are the mean values of the posterior parameter distributions.

Parameter name	Symbol	Original Value (11)	Prior distribution minimum	Prior distribution maximum	Posterior distribution mean	Source(s)
Rate of recovery from colonization	ρ_C_	12.175	8.35	11.48	10.59	[13, 14]
Rate of recovery from disease	ρ_D_	36.5	30.26	46.7	36.13	[15]
High protection against colonization	α_H_	75%	75%	95%	91%	[16]
Low protection against colonization	α_L_	25%	25%	70%	60%	[11]
High protection against disease	β_H_	100%	75%	100%	88%	[17, 18]
Low protection against disease	β_L_	90%	50%	95%	77%	[19]
Rate of waning from high to low protection, by age:	ω_H_(a)					
<6 months		0.57	0.26	1.04	0.65	[19]
6 months– 2 years		0.341	0.26	0.52	0.39	[17, 20]
3–10 years		0.275	0.26	0.52	0.39	[18, 20, 21]
≥11 years		0.05	0.10	0.26	0.18	[18, 20–22]
Rate of waning from low to no protection, by age:	ω_L_(a)					
<6 months		0.506	0.10	0.21	0.15	[19]
6 months– 2 years		0.254	0.03	0.05	0.04	[18, 20]
3–10 years		0.19	0.03	0.05	0.04	[18, 20]
≥11 years		0.03	0.005	0.05	0.02	[22]
Rate of waning from vaccinated to high protection, by age:	ω_V_(a)					
<6 months		[not eligible for vaccination]				
6 months– 2 years		0.009	Not estimated by ABC		0.069	[23]
3–10 years		0.005			0.069	[24]
≥11 years		0.016			0.069	[24]
Force of infection from outside the population	part of λ(t,a)	0.0005	5.22x10^-^6	5.22x10^-^5		[11]
Rate of disease among the colonized as x + y*age(years)	σ(a)					
Dry season x		0.0019	0.0965	0.102	0.099	[11]
Dry season y		-1.04x10^-5^	-4.70x10^-6^	-5.74x10^-6^	-5.21x10^-6^	[11]
Rainy season x		0.0018	0.00185	0.00195	0.099	[11]
Rainy season y		-1.1x10^-5^	4.70x10^-6^	5.74x10^-6^	-5.25x10^-6^	[11]
Vaccine effectiveness	VE	90%	Not estimated by ABC		90%	[8]

Our model was fit to data on the prevalence of NmA colonization in Burkina Faso. Burkina Faso census data were used to define the population starting size, age distribution, birth rates, and death rates. Other model parameters were defined as much as possible based on published data, as previously described (Table 1).[11] Estimates of the age-specific force of infection were not available in the literature. We therefore estimated age-specific effective contact rates (i.e., a “who acquires infection from whom” (WAIFW) matrix) using colonization prevalence data, with separate estimates for dry seasons vs. rainy seasons, to account for the seasonality of NmA in the meningitis belt.

We originally developed two versions of the model.[11] Our Base model assumed that PsA-TT vaccination was as effective as natural infection in preventing future colonization or disease; in this model, individuals move to the “High Protection” compartment after vaccination. We also created a Vaccination-Plus model in which PsA-TT vaccination is assumed to induce a stronger immune response than natural infection (not shown; see [11]); in that model, all vaccinated individuals move to a “Vaccinated” compartment. Individuals in the Vaccinated compartment are immune to both disease and colonization until protection wanes, at which point they move to the “High Protection” state.

### *A priori* model updates

We made several modifications to our model, based in part on new data that have emerged since 2010. First, in developing our initial model, we used waning of SBA against serogroup C following vaccination with monovalent (MenC) or quadrivalent (MenACWY) vaccine as proxies for waning of high and low protection levels in our model. Specifically, we had assumed that duration of MenC SBA titers ≤1:128 following MenC or MenACWY vaccination corresponds to duration of High Protection, and duration of MenC SBA titers ≤1:8 corresponds to duration of Low Protection. In our Vaccination-Plus model, we had assumed that duration of NmA SBA titers ≤1:128 following PsA-TT vaccination corresponds to the duration of the Vaccinated state. After we developed our model, additional data have been published on these waning rates, including PsA-TT antibody persistence data.[20, 22–24] Taken together with the prior studies,[17, 18, 21] these data suggest faster rates of High/Low and Low/None waning in certain age groups than we originally assumed (Table 1). We included these updated data in refitting our model parameters, as described below.

Second, the model includes a stochastic term that modifies the force of infection by a percentage of the base value. This creates recurring but irregular epidemics, similar to observed disease patterns. In our original model, we resampled this stochastic term monthly. This required the stochastic term to have a wide range (±75%) to reproduce observed disease patterns. We updated the model to resample the stochastic term yearly, which may be more biologically plausible, reflecting annual variation in climate or other external factors.[25] With this change, the stochastic term covers a much smaller range (±20%, drawn from a uniform distribution) to reproduce observed epidemic patterns.

Finally, as originally implemented, our Vaccination-Plus model included an assumption of sterilizing immunity, in that vaccination of colonized individuals resulted in a loss of colonization. Here, we update the Vaccination-Plus model so that colonized individuals do not lose colonization upon vaccination (Fig 1B).

To incorporate newly available data into our model, we refit the model parameters to observed data on colonization and disease prior to the use of PsA-TT. Target incidence data were as previously described.[11] For target colonization data, we combined prior estimates of the prevalence of NmA colonization by season in Burkina Faso[26] with a recent meta-analysis of age-specific colonization of Nm in Africa.[27] In our prior work, we used fixed values for most model parameters based on the literature and estimated the WAIFW matrix and per-carrier rate of invasive disease from colonization and incidence data using an interative numeric algorithm. In our updated work, to better capture uncertainty in all the model parameters, we moved to the Bayesian paradigm. We set prior distributions for all model parameters based on the existing literature, using uniform distibutions (Table 1). We estimated posterior distributions for all parameters using approximate Bayesian computation (ABC).[28] In brief, we summarized the observed data based on three metrics: age-specific prevalence of colonization by season, age-specific incidence of invasive disease, and the frequency of major epidemics. We then ran 200,000 iterations of our simulation model, where parameters values were randomly sampled from the prior distributions before each iteration. In each iteration, we compared simulated to observed data on each metric. Parameter sets where simulated data matched observed data within pre-defined tolerance levels were accepted, while the rest were rejected. The accepted parameter sets form the posterior distributions.

To reduce the number of parameters to estimate, we made some simplifying assumptions to the WAIFW transmission matrix. First, given that age-specific colonization prevalence appears to follow the same pattern during dry and rainy seasons,[27] we did not estimate separate WAIFW matrices for dry and rainy seasons. Rather, we assumed that the rainy season matrix was a proportional reduction in the dry season matrix, and estimated a single parameter to identify that proportion. Second, we constrained the number of unique beta values (i.e. effective contact rates) across selected cells of the WAIFW matrix. Finally, to increase the flexibility of the simplified WAIFW matrix, we expanded it to five age groups (<5 years, 5–9 years, 10–14 years, 15–19 years, ≥20 years) rather than four as originally used (Table 2).

**Table 2:** Mean posterior values for who acquires infection from whom matrix (i.e. effective contact rates per year by age group).

		Age of infectious contact (years)				
		<5	5–9	10–14	15–19	≥20
Age of susceptible contact (years)	<5	1.81	1.81	1.81	1.81	1.81
	5–9	3.89	3.89	3.89	3.89	3.89
	10–14	6.12	6.12	8.17	6.12	6.12
	15–19	6.79	6.79	6.79	11.73	6.79
	≥20	5.13	5.13	5.13	5.13	5.13

### Incidence data 2011–2017

Clinically suspected and confirmed NmA meningitis from 2001–2017 were obtained through national, population-based meningitis surveillance systems.[29] The annual number of clinically suspected meningitis cases was determined from the aggregate meningitis surveillance system, in which the number of suspected meningitis cases and deaths are transmitted weekly from the district to the national level. The number of confirmed NmA cases was determined from the case-based meningitis surveillance system, in which detailed epidemiological and laboratory data are collected on each suspected meningitis case. We imputed total annual NmA cases based on the suspected meningitis cases and the proportion of specimens that tested positive for NmA.

### Analysis

To identify which assumptions best fit the observed incidence of NmA in Burkina Faso following the introduction of PsA-TT, we simulated NmA incidence using four models: the original Base model from Tartof et al[11] (“Original Base”), the Vaccination-Plus model of Tartof et al (“Original Vaccination-Plus), the Base model from the updated model fitting (“Updated Base”), and the Vaccination-Plus model from the updated model fitting (“Updated Vaccination-Plus”). We ran 500 iterations of each model. For each iteration, after a 50-year burn-in, we calculated the annual simulated incidence of invasive NmA in Burkina Faso during the period 2001–2017. In each year of this period, we calculated the mean and standard error of the yearly incidence from the 500 model iterations. We quantitatively assessed whether the simulated mean significantly differed from the observed cases during 2001–2017 and qualitatively assessed whether the simulated mean was capturing the observed trends.

After identifying the model that best reproduced observed trends in NmA disease following vaccination, we used that model to forecast the impact of five potential vaccination scenarios during the period 2011–2050:

- a “campaign only” scenario, in which no further vaccines are given after the 2010 mass vaccination campaign of persons aged 1–29 years;
- a “campaign plus EPI” scenario, in which routine PsA-TT vaccination at 9 months of age is introduced 5 years after the campaign;
- a “periodic mini-campaign” scenario, in which children aged 1–4 are vaccinated in catch-up campaigns conducted every 5 years;
- a “campaign, catch-up, EPI,” in which the initial campaign is followed by a five-year gap, at which point there is a catch-up campaign for children 1–4 years of age, and then routine EPI vaccination.

In all scenarios we assumed 100% coverage of the initial campaign[7] and 80% vaccine coverage for both routine vaccination and for catch-up campaigns.[30]

### Sensitivity analysis

Recent studies on the waning of SBA titers following PsA-TT vaccination show persistence of high reference strain SBA titers up to five years post-vaccination, suggesting that PsA-TT confers long-term protection against NmA colonization and disease.[23, 24] However, some studies suggest that SBA titers against the vaccine reference strain may not correlate with clinical protection against other NmA strains, and alternate serologic indicators of vaccine-induced immunity may be needed.[31, 32] If vaccine-strain SBA does not cross-protect against other strains, VE could decrease over time as new NmA strains emerge. To allow for this possibility, we repeated all analyses with the Updated Vaccination-Plus model assuming fast waning from the Vaccinated state (mean duration of protection, 4.6 years).

Secondly, as a potential response in light of fast antibody waning, we included a fifth possible vaccination scenario with the fast-waning Updated Vaccination-Plus model, dubbed “campaign, EPI, booster,” in which a booster dose is offered to at 10 years of age to children who received PsA-TT as infants. We assumed 70% coverage of the 10-year booster dose.

Finally, using the Updated Vaccination-Plus model, we repeated the “campaign plus EPI” analyses, assuming 60% EPI coverage rather than 80% in the base case.

## Results

### Fit of updated model to observed patterns

**Fig 2:**
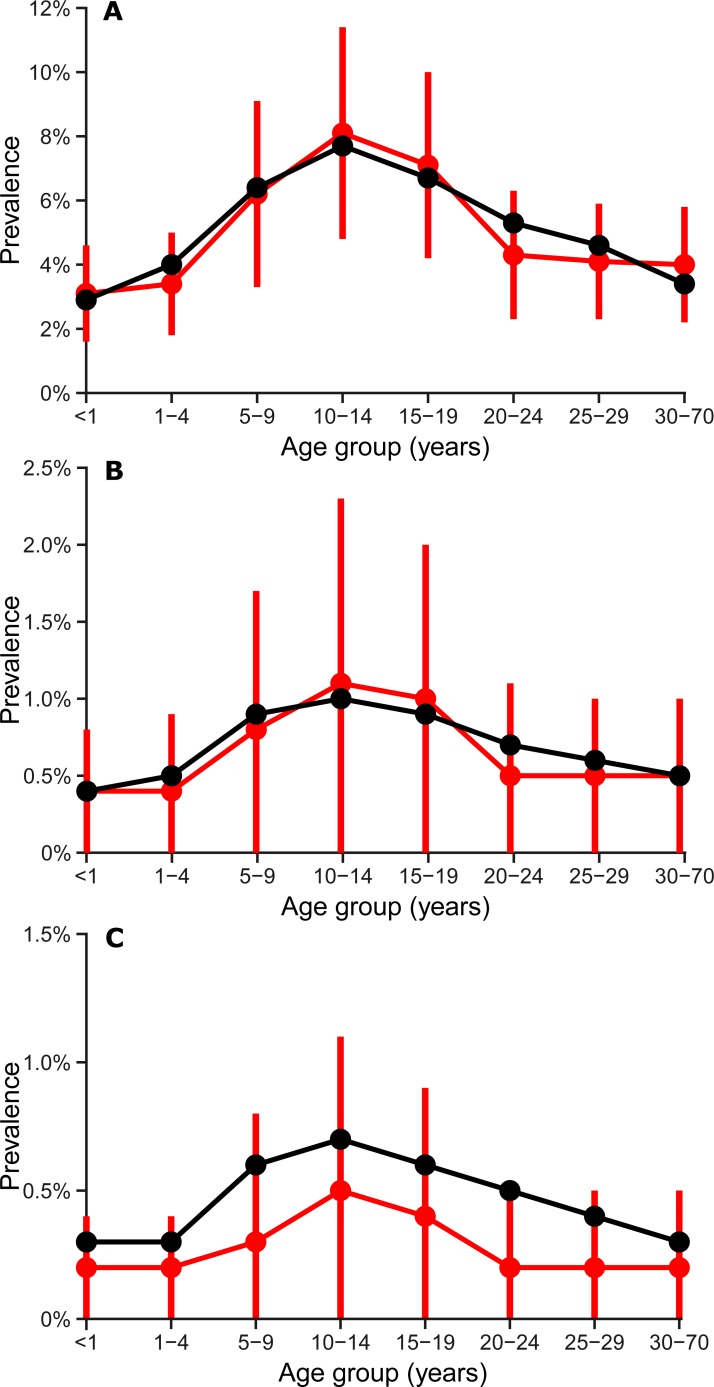
Comparison of observed (black) and simulated (red) prevalence of NmA colonization by age and season–Burkina Faso. A) Dry seasons with major epidemics; B) Dry seasons without major epidemics; C) Rainy seasons. Dots indicate means, and vertical red lines represent standard deviations of simulated prevalence across 100 simulation iterations.

**Fig 3:**
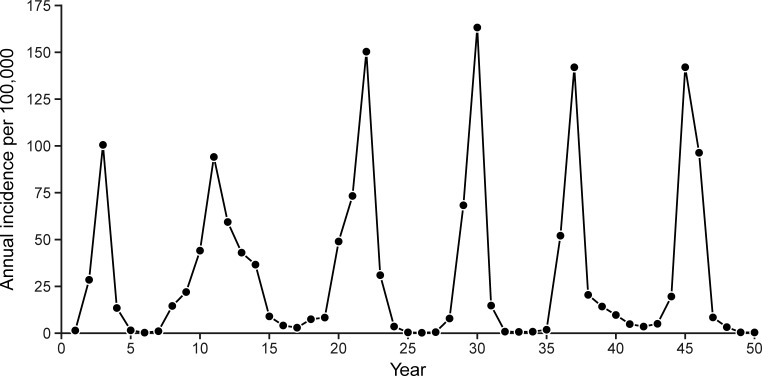
Simulated annual incidence of invasive NmA from a typical simulation run over a 50-year period assuming no vaccination—Burkina Faso.

The updated model (with updated WAIFW matrix, Table 2) was able to re-create the key dynamics of meningococcal disease in Burkina Faso. The simulated age-specific prevalence of colonization were well-matched to observed data (Fig 2, r = 0.99), as was the fit to age-specific incidence (r = 0.97). Simulated incidence was characterized by major epidemics that occurred a median of 8 years apart (interquartile range, 6 to 11 years), some of which persisted across multiple dry seasons (Fig 3).

### Comparing observed and simulated cases, 2001–2017

**Fig 4:**
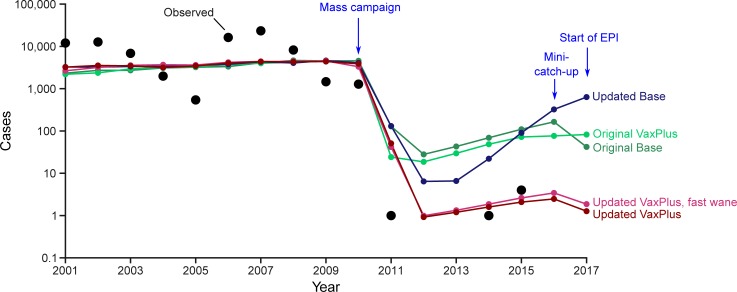
Observed and simulated NmA cases assuming mass vaccination campaign in December 2010, catch-up in 2016, and EPI beginning in 2017—Burkina Faso, 2001–2017. Simulated cases represent mean incidence across 500 simulation iterations. Zero cases were observed during 2012, 2013, 2016, and 2017; zero cases not plotted due to use of log scale.

From 1998 through 2010, a mean of 6,772 NmA cases occurred annually among residents of Burkina Faso, with major epidemics during 2001–2002 and 2006–2007. After the vaccination campaign in December 2010, cases dropped by more than 99.9%: one NmA case was detected in 2011, one in 2014, four in 2015, and zero in 2012, 2013, 2016, and 2017 (Fig 4). In all four models, simulated case counts dropped more than 97% in 2011 following the 2010 vaccination campaign (Fig 3). In the Original Base and Original Vaccination-Plus models, the mean incidence following vaccination never fell below 10 cases per year, and quickly rose to 124 or 81 cases (respectively) by 2016. The Updated Base model achieved significantly fewer cases than either Original model (p < 0.05) in 2012–2015, but also resulted in increasing cases in 2015–2017, which was not observed in practice.

In contrast, the Vaccination-Plus model resulted in a mean of 1–2 cases per year between 2012 and 2017, significantly lower than the other three models (p < 0.05) and very close to the observed case counts. In sensitivity analysis that assumed fast waning of vaccine-induced protection for the Updated Vaccination-Plus model, simulated incidence was slightly higher (1–3 cases per year), but not significantly so (p > 0.05).

As with the other models, the Updated Vaccination-Plus model predicted significantly more cases in 2011 (30) than were observed (1) (p < 0.05). This is partially an artefact of the stochastic simulations. The 500 iterations vary in the timing of major epidemics, while the actual history of NmA in Burkina Faso included a major epidemic in 2006–2007 but not thereafter. If we restrict the simulations to those with a major epidemic in 2007 (at least 15,000 cases), this set of simulations resulted in a mean of 6 cases predicted for 2011.

### Relative effectiveness of vaccination scenarios

**Fig 5:**
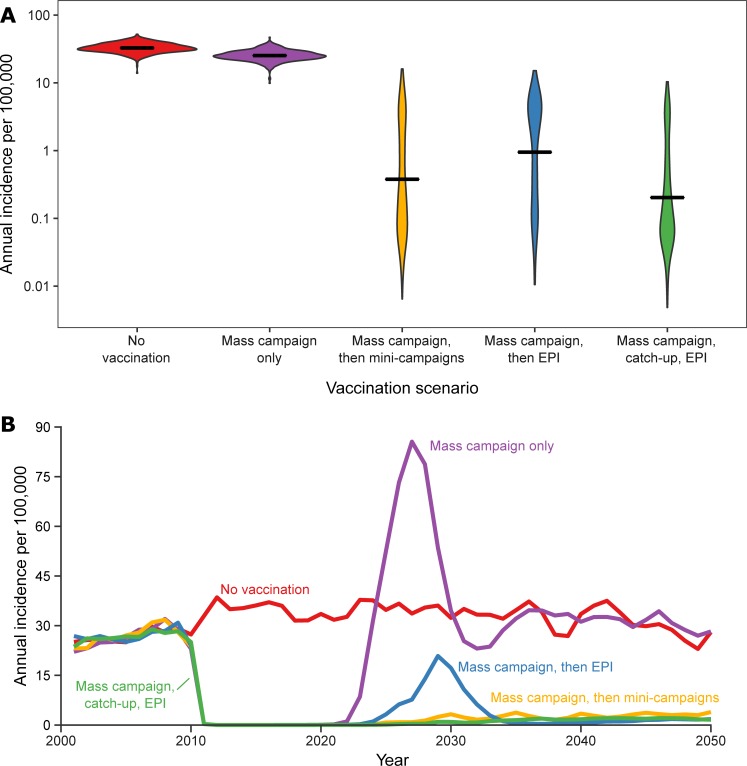
Annual incidence of invasive NmA disease per 100,000 population under five possible vaccination scenarios–Burkina Faso, 2011–2050. A) Density plot of predicted annual incidence under each scenario for 500 simulations; B) Mean annual incidence across simulations under each scenario.

We forecast the effects of four different vaccination programs using the Updated Vaccination-Plus model. All four hypothetical vaccination programs are predicted to reduce disease incidence over the time period from 2011–2050, relative to no vaccination. Using only a mass campaign in 2010 results in an average annual incidence of 25.7 cases per 100,000 population (Fig 5A) over that time, with most of the decrease due to low case counts during the first 15 years after the mass campaign, after which NmA disease resurges and returns to pre-campaign levels (Fig 5B). The three other programs are predicted to yield sustained reductions in NmA disease, with mean annual incidence ranging from 0.9 (campaign, catch-up, EPI) to 2.7 (campaign plus EPI) cases per 100,000. These three programs have essentially identical incidence at equilibrium, but the “campaign plus EPI” is projected to have a slight resurgence of NmA disease approximately 20 years after the vaccination campaign. This is due to the five-year lag between the initial mass campaign and the initiation of EPI vaccination, which leaves a cohort of children who lack immunity to NmA colonization. If EPI is initiated immediately after the mass campaign (data not shown), or with a mini-catch-up campaign prior to EPI (Fig 5), this resurgence does not occur.

### Sensitivity analyses

In sensitivity analyses that assumed fast waning of protection, using only a mass campaign in 2010 resulted in an average annual incidence of 26.5 cases per 100,000 population between 2011 and 2050. In comparison, all four long-term vaccination programs are predicted to yield sustained reductions in NmA disease. Mean annual incidence ranged from 3.1 (campaign, EPI, booster) to 8.3 (campaign plus EPI) cases per 100,000. The relative ranking of “campaign plus EPI”, “campaign, catch-up, EPI”, and “periodic mini-campaigns” was the same in the sensitivity analyses as in the main analyses. In this setting, the “campaign, EPI, booster” program was projected to have the lowest long-term incidence (p < 0.05 for comparison with each other program). When comparing “campaign plus EPI” at 60% vs. 80% EPI coverage, long-term annual incidence was significantly higher at 60% coverage (mean, 8.1 cases per 100,000) compared to 80% coverage (mean, 2.7 cases per 100,000) (p = 0.003).

## Discussion

Mathematical and simulation models support public health decision making in a variety of contexts, such as comparing the relative merits of different potential vaccination programs (e.g.,[33–35]). The ongoing process of model validation, in which model predictions are compared against actual events, increases a model’s credibility and thus its utility to decision makers.[36, 37] In this paper, we updated an existing model of NmA disease in Burkina Faso developed prior to PsA-TT implementation in light of newly available data on duration of PsA-TT immunity. We then compared our original and updated models against observed incidence of NmA disease in Burkina Faso following implementation of PsA-TT vaccination. In previous work, conducted prior to the first PsA-TT vaccination campaign in Burkina Faso, we had uncertainty about the duration of vaccine-induced immunity relative to immunity following natural infection. We incorporated this uncertainty by using two different model structures–our “Base” and “Vaccination-Plus” models. One goal of this present study was to resolve this structural uncertainty. We found that the updated Vaccination-Plus model, in which vaccination confers greater protection against NmA than natural disease, most accurately reproduced the epidemiology of NmA disease.

When using our best-fit model, both post-campaign strategies (adding PsA-TT to the EPI schedule or conducing periodic campaigns among young children) were predicted to result in long-term reductions in NmA incidence relative to no vaccination or to a mass campaign alone. Over a 40 year time span, the average difference between the strategies was small (approximately 1 case per 100,000 per year). However, adding PsA-TT to the EPI schedule with a five year lag was predicted to lead to a temporary resurgence of cases roughly 20 years after vaccine. In contrast to our findings, a NmA model developed by Karachaliou and colleagues,[25] which suggests that adding PsA-TT to the EPI schedule would be slightly more beneficial over a 40-year period (roughly 1.5 fewer cases per 100,000 per year) than periodic campaigns. As the two models make different assumptions regarding model structure and certain parameters, it is not surprising to find that the models differ on the relative ranking of these two strategies, particularly given the small relative differences between the strategies in each model. One possible explanation is the fact that Karachaliou et al used partial differential equations, with continuous calendar time and continuous aging, while we used partial difference equations with discrete calendar time (1 week time steps) and age groups (1 month). Simulations by Karachaliou suggest that use of continuous aging may slightly favor EPI vaccination compared to discrete aging (A. Karachaliou, personal communication).

Several limitations of our model must be considered. First, our model only considers colonization and disease due to serogroup A *N*. *meningitidis*. It is possible that reductions in NmA colonization following PsA-TT vaccination may create an ecologic niche that could be filled by hypervirulent *N*. *meningitidis* strains of a different serogroup (such as serogroup W [38] or serogroup C [39]). This serogroup replacement would reduce the long-term gains of PsA-TT vaccination on overall numbers of suspected meningitis cases relative to our forecasts. Predicting vaccine effects in the presence of potential serogroup replacement would require a multi-strain *N*. *meningitidis* model. Second, our model assumes that the vaccine maintains its current effectiveness over the long term. As the duration of protection of PsA-TT remains unknown, if vaccine effectiveness wanes more substantially than predicted based on waning of reference strain SBA, the model could overestimate reductions in disease due to vaccination.

The findings of these models highlight the critical need for long-term immunization strategies in order to sustain the gains made by mass PsA-TT vaccination on the elimination of NmA epidemics in sub-Saharan Africa.[9, 10] Mass vaccination campaigns alone in the absence of subsequent introduction into routine EPI or periodic catch-up campaigns could lead to devastating epidemics within 15 years of the campaign. Mathematical models suggest that routine PsA-TT vaccination as part of the EPI program an effective strategy for maintaining low incidence of NmA disease in the wake of mass campaigns if initiated immediately after the mass campaign or if followed by a catch-up campaign for children missed in the initial campaign.

Disclaimer: The findings and conclusions in this report are those of the authors and do not necessarily represent the official position of the Centers for Disease Control and Prevention.
